# Supplementary figures and images for: No evidence for an association of plasma homocysteine levels and refractive error – Results from the population-based Gutenberg Health Study (GHS)
Source: PLoS One. 2020 Apr 13;15(4):e0231011. doi: 10.1371/journal.pone.0231011 (PMC7153866; doi:10.1371/journal.pone.0231011)

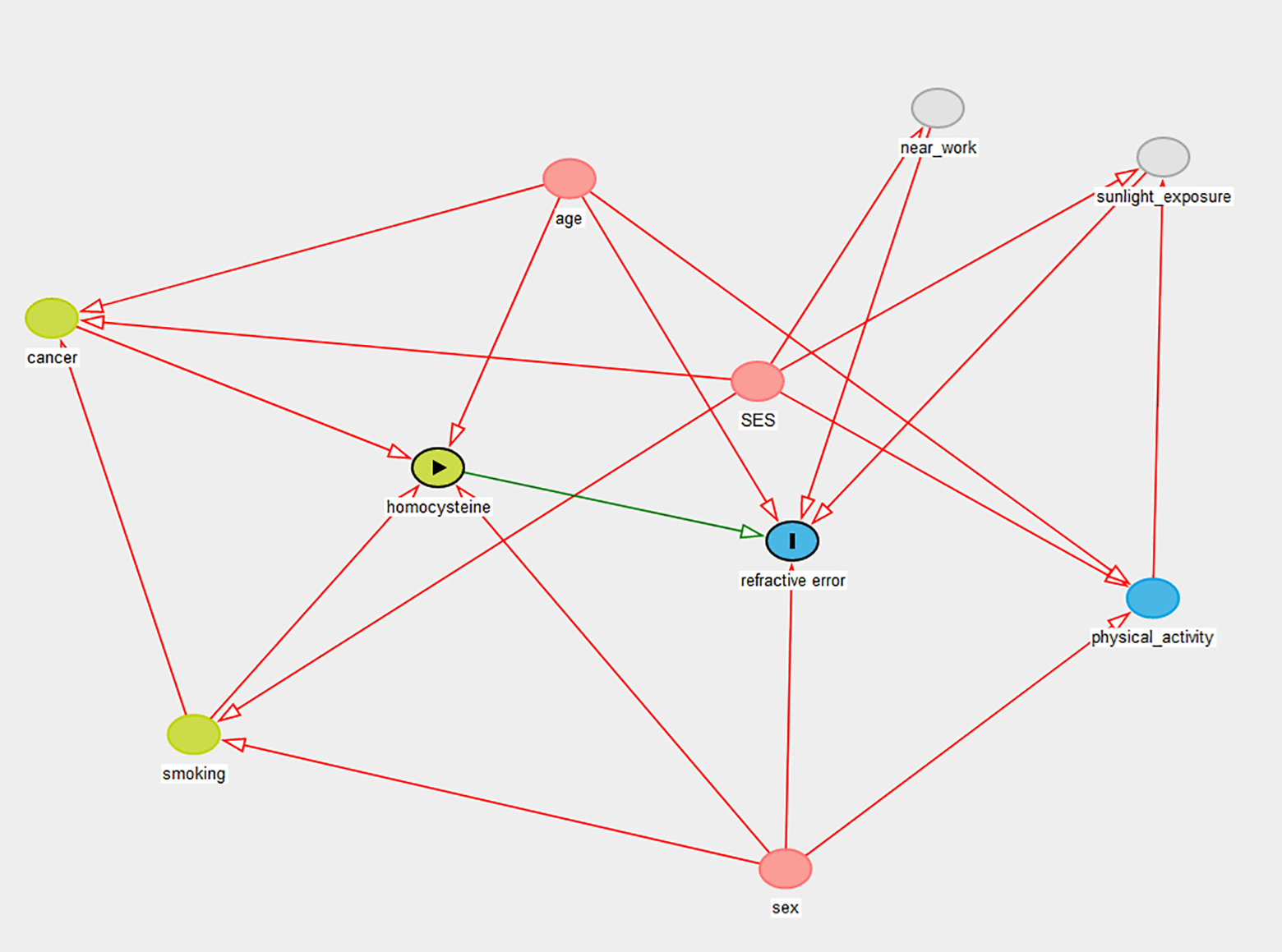

Supplement: S1 Fig — Generated with DAGitty 2.3 (http://dagitty.net/, last accessed 2019-08-19). SES = socio-economic status (TIF) [file pone.0231011.s001.tif]

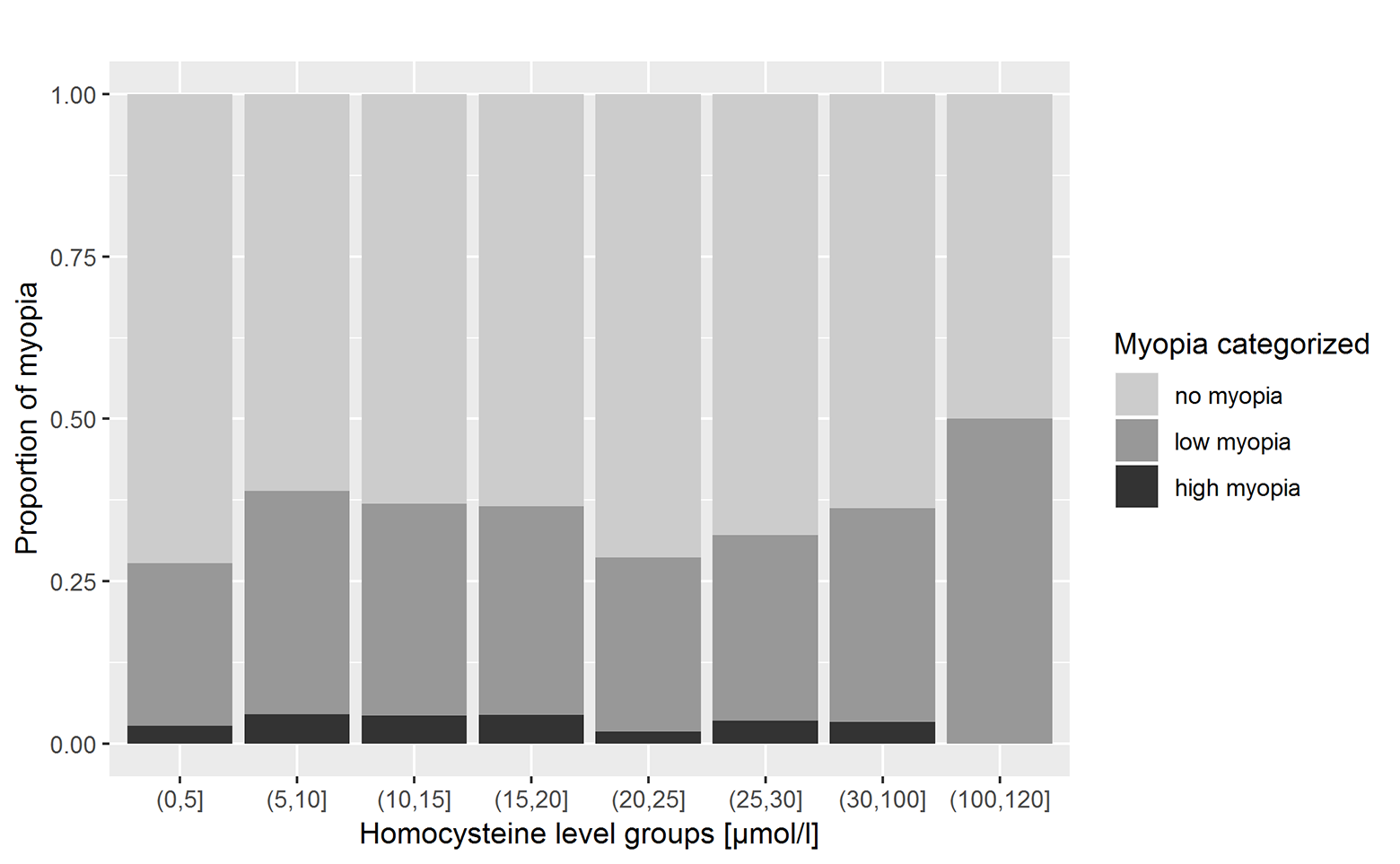

Supplement: S2 Fig — Low myopia: spherical equivalent < = -0.5 D—>-6 D in either eye; high myopia: spherical equivalent < = -6 D in either eye, no myopia: spherical equivalent > -0.5 D in both eyes. N included in homocysteine categories (left to right): 36, 4836, 6942, 1484, 272, 84, 91, 4. (TIF) [file pone.0231011.s002.tif]

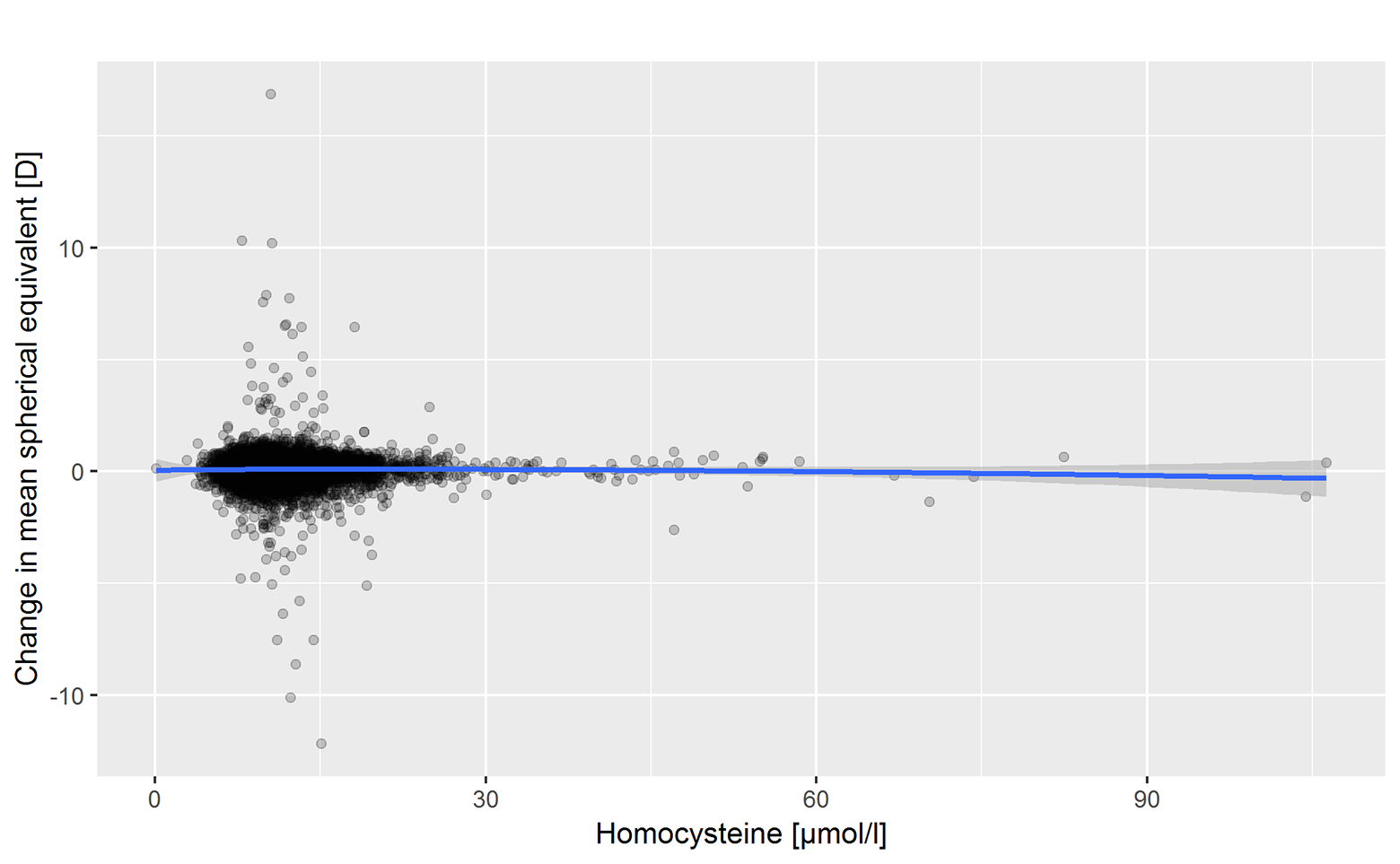

Supplement: S3 Fig — Smoothing line with 95% confidence bands based on locally weighted scatterplot smoothing (LOESS). (TIF) [file pone.0231011.s003.tif]

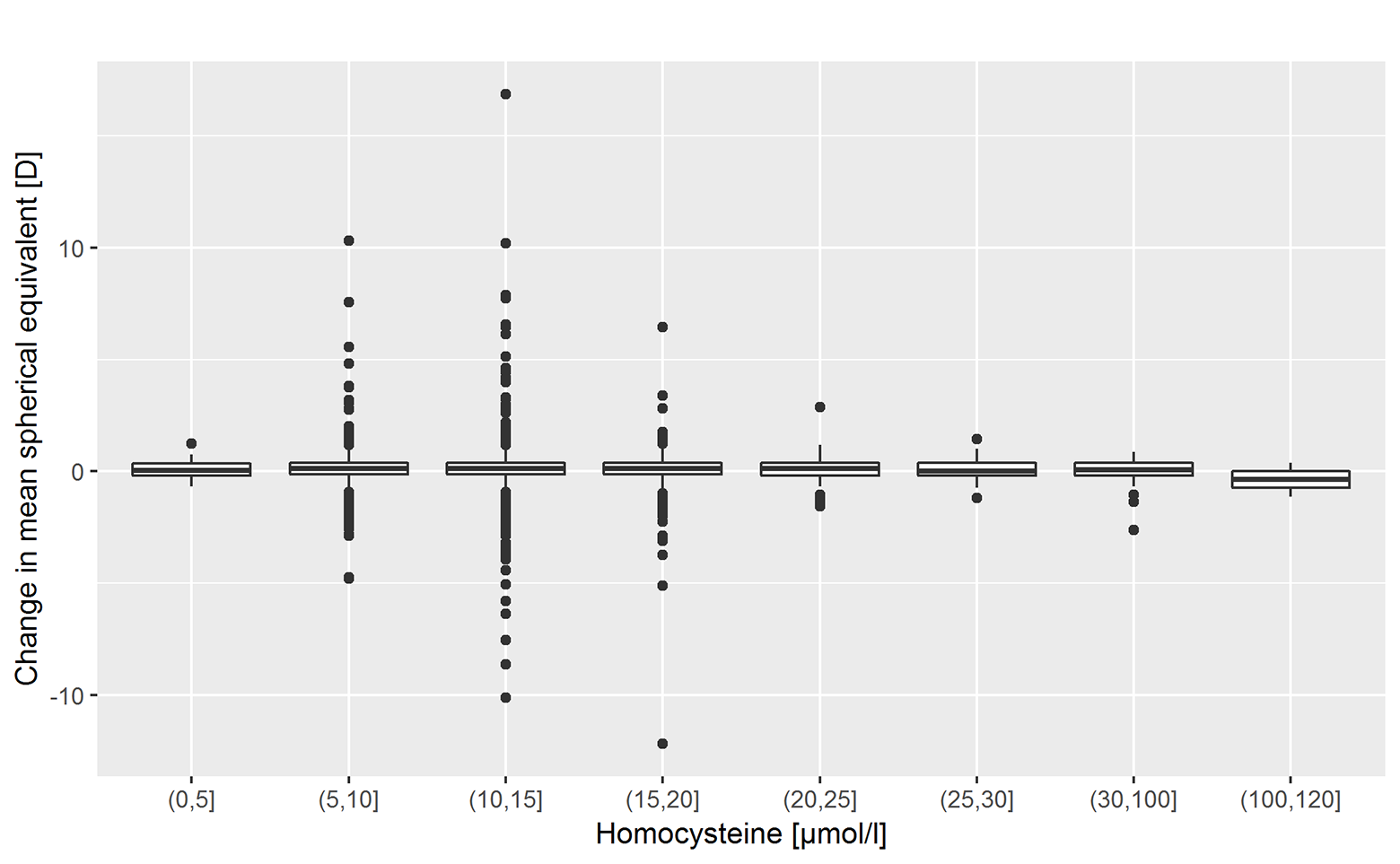

Supplement: S4 Fig — N included in homocysteine categories (left to right): 36, 4836, 6942, 1484, 272, 84, 91, 4. (TIF) [file pone.0231011.s004.tif]
