# Supplementary material for: No evidence for an association of plasma homocysteine levels and refractive error – Results from the population-based Gutenberg Health Study (GHS)
Source: PLoS One. 2020 Apr 13;15(4):e0231011. doi: 10.1371/journal.pone.0231011 (PMC7153866; doi:10.1371/journal.pone.0231011)
Supplement: S1 Table — (PDF) [file pone.0231011.s005.pdf]

S1 Table: Baseline characteristics of the German population-based Gutenberg Health Study (GHS) subsample for the analysis of 5-year change in refraction in relation to baseline homocysteine levels.

	**Overall (n=9,928)**	**Men (n=5,115, 51.5%)**	**Women (4,813, 48.5%)**
Age [years]	53.28 (10.45)	53.56 (10.55)	52.99 (10.34)
Socio-economic status	13.00 [10.00, 17.00]	14.00 [10.00, 18.00]	12.00 [10.00, 16.00]
Hypertension	4580 (46.2)	2645 (51.7)	1935 (40.2)
Diabetes mellitus	675 (6.8)	441 (8.6)	234 (4.9)
Dyslipidemia	3205 (32.3)	2110 (41.3)	1095 (22.8)
Obesity (BMI>=30)	2310 (23.3)	1258 (24.6)	1052 (21.9)
Smoking	1862 (18.8)	1011 (19.8)	851 (17.7)
Self-reported cancer	753 (7.6)	343 (6.7)	410 (8.5)
Sphere (OD) [diopter]	-0.18 (2.42)	-0.19 (2.34)	-0.16 (2.51)
Sphere (OS) [diopter]	-0.19 (2.44)	-0.22 (2.40)	-0.15 (2.49)
Cylinder (OD) [diopter]	-0.55 (0.65)	-0.57 (0.67)	-0.52 (0.62)
Cylinder (OS) [diopter]	-0.54 (0.63)	-0.56 (0.65)	-0.51 (0.60)
Spherical equivalent (OD) [diopter]	-0.45 (2.45)	-0.48 (2.36)	-0.43 (2.55)
Spherical equivalent (OS) [diopter]	-0.46 (2.47)	-0.50 (2.42)	-0.41 (2.52)
Visual acuity (OD) [logMAR]	0.00 [0.00, 0.10]	0.00 [0.00, 0.10]	0.00 [0.00, 0.10]
Visual acuity (OS) [logMAR]	0.00 [0.00, 0.10]	0.00 [0.00, 0.10]	0.00 [0.00, 0.10]
Homocysteine [µmol/l]	10.90 [9.20, 13.10]	11.90 [10.20, 14.00]	10.00 [8.40, 11.90]

For categorical variables: absolute (relative) frequencies; for continuous variables: mean (standard deviation), in case of skewed distribution median (25th/ 75th percentile).
