# Supplementary material for: No evidence for an association of plasma homocysteine levels and refractive error – Results from the population-based Gutenberg Health Study (GHS)
Source: PLoS One. 2020 Apr 13;15(4):e0231011. doi: 10.1371/journal.pone.0231011 (PMC7153866; doi:10.1371/journal.pone.0231011)
Supplement: S2 Table — (PDF) [file pone.0231011.s006.pdf]

S2 Table. Distribution homocysteine levels by myopia categories in the baseline sample of the German population-based Gutenberg Health Study (GHS), 2007-2012.

	**all**		**Men**		**Women**	
	n	Homocysteine [µmol/l]: median (25th/ 75th percentile)	n	Homocysteine [µmol/l]: median (25th/ 75th percentile)	n	Homocysteine [µmol/l]: median (25th/ 75th percentile)
**no myopia**	8609	11.10 [9.40, 13.40]	4310	12.00 [10.30, 14.40]	4299	10.20 [8.70, 12.20]
**low myopia**	4540	11.00 [9.10, 13.20]	2368	12.00 [10.20, 14.20]	2172	9.90 [8.30, 11.80]
**high myopia**	600	11.00 [9.20, 13.10]	297	12.10 [10.30, 14.10]	303	9.90 [8.45, 11.95]

Low myopia: spherical equivalent <=-0.5 D - >-6 D in either eye; high myopia: spherical equivalent <= -6 D in either eye, no myopia: spherical equivalent > -0.5 D in both eyes.
