# Supplementary material for: No evidence for an association of plasma homocysteine levels and refractive error – Results from the population-based Gutenberg Health Study (GHS)
Source: PLoS One. 2020 Apr 13;15(4):e0231011. doi: 10.1371/journal.pone.0231011 (PMC7153866; doi:10.1371/journal.pone.0231011)
Supplement: S3 Table — (PDF) [file pone.0231011.s007.pdf]

S3 Table. Association of homocysteine levels (per 10 µmol/l]) with diopters of spherical equivalent (sensitivity analyses) – Results from the German population-based Gutenberg Health Study (GHS).

	**Univariate**		**Adjusted for age and socio-economic status**	
	**Estimate (CI)**	**p-value**	**Estimate (CI)**	**p-value**
**In men only (n=6,945)**	0.08 (0.00 - 0.16)	0.05	-0.06 (-0.13 - 0.02)	0.14
**In women only (n=6,729)**	0.43 (0.32 - 0.55)	2.7E-13	-0.01 (-0.12 -0.10)	0.84
**In participants with hyperhomocysteinemia >15 µmol/l (n=1,921)**	0.02 (-0.09 - 0.12)	0.72	0.03 (-0.07 - 0.13)	0.55
**In participants without severe astigmatism (both eyes cylinder > -1 diopter, n=7,198)**	0.06 (-0.02 - 0.14)	0.14	-0.09 (-0.18 - -0.01)	0.02

Results from linear regression, using generalized estimating equation models to account for the correlation of fellow eyes, p value derived by Wald score test.
