# Supplementary material for: No evidence for an association of plasma homocysteine levels and refractive error – Results from the population-based Gutenberg Health Study (GHS)
Source: PLoS One. 2020 Apr 13;15(4):e0231011. doi: 10.1371/journal.pone.0231011 (PMC7153866; doi:10.1371/journal.pone.0231011)
Supplement: Attachment — Submitted filename: reviewers_comments_20190902.docx [file pone.0231011.s008.docx]

Dear Editor,

we are thankful for the constructive comments and suggestions raised by the reviewers. We have thoroughly worked through the manuscript and have answered the specific comments point-by-point.

We hope that our revised manuscript will be feasible for publication in “PLOS ONE”.

## Reviewer #1:

**This paper reports that there is no systematic association between refractive status and levels of homocysteine in the adult sample involved in the GHS. In a paper of this kind, with negative results, it is important to establish that the hypothesis being tested was plausible in the first place, since it is possible that, in a complex study such as the GHS, many implausible or arbitrary hypotheses could be postulated. However, the case that homocysteinemia is associated with myopia is strong, and therefore the hypothesis that refractive status is affected by less extreme levels of homocysteine is plausible, and worth testing. The failure to find any statistical association of homocysteine levels with refractive categories is clear in the results. Given that the sample size is large, the conclusion drawn seems reasonable.**

- **However, there are some aspects of the paper which require attention. Firstly, it is not clear if the criteria for inclusion in the GHS would have excluded homocysteimemia. This should be explained. Since this is a rare condition, even with the large sample size of the GHS, perhaps only 1 or 2 cases would have been expected.**
  There were no other exclusion criteria than the criteria mentioned in the M&M section:
  “Exclusion criteria were insufficient knowledge of German and physical or mental inability to participate in the examinations in the study center. […] For the analysis of refraction, we included all participants with available information on Hcy and refraction at baseline and with phakic lens status and no history of corneal surgery or corneal laser treatment. For the analysis of 5-year change in refraction, we further excluded all participants without Hcy or refraction measurements at the 5-year follow-up examination and with lens replacement surgery, corneal surgery or corneal laser treatment between baseline and follow-up examination.”
- **In the paper, the authors have largely compared the levels of homocysteine between refractive categories. However, there is another way to cut the data, as shown in Figures 1 and 2, looking at the how spherical equivalent varies depending on homocysteine levels. The analysis of this data is not taken very far, but if bins of homocysteine levels were considered, eye-balling the data would suggest that higher levels of homocysteine are likely to be associated with higher rather than lower levels. In some ways, I find this a more powerful way of looking at the data, given that it makes homocysteine levels the equivalent of an independent rather than a dependent variable.**
  We added the requested boxplots by bins of homocysteine levels (whish that was my idea…).

### Detailed comments:

- **The authors should explain why they have chosen to use a cut-off for no myopia of >-0.75D. This is an unusual cut-off, which would include people with a refraction of -0.5D who would be classed as myopic in most studies.**
  We changed the cut-off, according to a recent publication:
  “The spherical equivalent (SE) was calculated as the spherical correction value plus half the cylindrical power. Participants were then categorized by their SE into having low myopia (<=-0.5 D - >-6 D in either eye), high myopia (<=-6D in either eye), or no myopia (>-0.5 D in both eyes), following the recent International Myopia Institute (IMI) proposition [11].”
- **In the Introduction, the authors cite the article by Dolgin on the evidence for genetic and environmental factors. While, this article has been very influential in bringing the evidence about myopia to a very general audience, I do not believe that it is appropriate to cite this article by a science journalist, as though it was a peer-reviewed analysis of the literature. Much of it reports on what some scientists think, with only a little evidence. To cite a proper scientific review that has been subject to peer-review is therefore important.**
  We deleted the Dolgin reference, and now include the following references:
  1.	Foster PJ, Jiang Y. Epidemiology of myopia. Eye (London, England). 2014;28(2):202-8. Epub 2014/01/11. doi: 10.1038/eye.2013.280. PubMed PMID: 24406412; PubMed Central PMCID: PMCPmc3930282.
  2.	Warner N. Update on myopia. Current opinion in ophthalmology. 2016;27(5):402-6. Epub 2016/05/27. doi: 10.1097/icu.0000000000000292. PubMed PMID: 27228418.
- **In line 116, the word “below” should be changed. In English writing, that means that something will follow in the text. In this case, I think the authors mean something like “For lower levels of visual acuity.”**
  ok
- **Line 125: A comma is needed after interview.**
  ok
- **Under Statistical Analysis, it is not clear why the authors used the regression strategy outlined. Regression analysis should be hypothesis-driven, rather than just an arbitrary inclusion of variables. I also find it useful to give a table of univariate associations, to give some context to adjusted results. In the context of this paper, it is not clear to me why the authors adjusted for diabetes mellitus, and for smoking, socioeconomic status, and education.**
  We changed the selection of confounders:
  “Model 1 included no covariates, model 2 was adjusted age, sex and socio-economic status. For the identification of potential confounders we followed the directed acyclic graph (DAG) concept [16]. We used the online tool DAGitty V2.3 (http://dagitty.net/, last accessed 2019-08-16) to visualize potential causal relationships (Supplementary Figure S) and to derive the minimal sufficient adjustment set for estimating the total effect of Hcy on refraction [17].”
- **Lines 156-157, the values for the prevalence of myopia are different in the Abstract. Why?**
  Corrected.
- **Lines 203-209 do not provide convincing arguments against the idea that the failure to measure cycloplegic refractions is a problem. The fact that associations with education were detected does not mean that weaker associations would be. Similarly, the argument that all subjects would have been affected in the same way is wrong. It is known that lack of cycloplegia leads to marked apparent myopic shifts in refraction for hyperopes, somewhat less shift for emmetropes and even less for myopes. Within these categories, there is also considerable variation. The fact is that this is a limitation, and its potential impact cannot be dismissed.**
  We follow the argument, and changed this part of the discussion:
  “A limitation of our study is the lack in precision in the measurement of refraction by using autorefraction without cycloplegia. There is evidence from epidemiologic studies that the resulting misclassification higher in, but not limited to children [19]. Furthermore, the misclassification is dependent on refractive error, our study outcome [20]. Therefore, there is the risk of a severe bias that might have diluted the effect we tried to explore. Despite this limitation, previous analyses, e.g. on the relationship between refraction and education, revealed that our measurement method is sufficiently precise to reveal associations [21], but this does not necessarily be the case for the subject of current analysis.”

## Reviewer #2:

**Nickels et al., investigated the association between concentrations of tHcy and myopia in a large cohort of healthy people from the area of Mainz, Germany. The hypothesis was that slightly elevated levels of tHcy are risk factor for myopia.it is not clear what slightly elevated is meant to be!**

**The study reported no association between these two factors in the studied cohort.**

**Considering the assumption of the authors and their motivation to conduct the present investigation (Line 80: Given the very strong association between severe hyperhomocysteinemia and myopia), a serious limitation of the present study is whether the right question was asked in the right cohort and dataset.**

**In a cohort with an age range between 35 and 74 years, it is very expected that myopia will be strongly related to age. Similarly HHCY will be driven by age. It is expected from studies in the field that an exposure threshold is investigated, since levels up to 12 or 14 µmol/ are considered normal and it is very unlikely for such an association (either weak or not present) to be detected within the normal range.**

**Sensitivity analysis is missing and some of the statistical analysis are not convincing in the present form. The discussion should include external validity and generalizability issues in the light of the tested hypothesis and results in this cohort.**

**The authors stated (line 148) that the study was exploratory. This means the expectations for an effect size were not concrete enough. However, the final conclusion is too absolute and states with a high confidence that: [We found no association between Hcy and myopia and so no evidence for a clinically significant contribution of minor elevations in Hcy to the risk of myopia], despite that the question can very likely not be answered with this data set.**

### Specific comments

- **The title states that tHcy was measured in serum, in the text (line 106) plasma homocysteine is referred to, but it is not mentioned which plasma was collected?**
  We changed the title, plasma is correct, and expanded the sampling/processing info:
  “Peripheral venous blood samples were obtained according to standard operation procedures, using a sampling container with a Hcy-stabilizing additive (“S-Monovette® HCY-Z-Gel”, Sarstedt AG & Co, Nürnberg, Germany), and were subsequently transported on ice to the main laboratory. After cooled centrifugation homocysteine was measured in plasma by an immunoassay on an Architect i2000SR analyzer (Abbott Diagnostics, Wiesbaden, Germany).”
- **2- Line 126-127: The socioeconomic status (SES) was defined based on income, education, and position. SES was divided into categories.**
  We deleted the information regarding SES categorization, since we did not use categories of SES in our analysis.
- **3- Statistical analysis: line 141, it is not clear what the primary and secondary variables should refer to.**
  We avoid these terms in the revised manuscript.
- **4- Statistical analysis: Line 145: in my opinion, sex is principally not considered a co-variate and the analysis should be performed separately for men and women. A confounder is defined based on the counterfactual thinking. Example: what would be the association or the risk in a given individual, should a given variable (e.g. diabetes mellitus) would not be present. The same argument can not apply for sex, since we have either men or women.**
  Following the reviewer’s comments, we revised the selection of potential confounders and the justification for our decisions in more detail. Different concept exist, we used the DAG concept by Greenland et al. (Greenland S, Pearl J , Robins JM . Causal diagrams for epidemiologic research. Epidemiology 1999;10:37–48.). I am not convinced that sex does not qualify for a confounder because sex could not be changed. In my opinion, sex could still distort the relationship if associated with both exposure and outcome, and if these conditions might be fulfilled, the analysis should be adjusted for sex. But I would be happy to discuss this further; maybe the reviewer can point me to a corresponding paper?
- **5- Statistical analysis: The variable education is already included in the socioeconomic status (model 3, and lines 126-127). There is not convincing reason to include education again as a co-variable in model 3. Otherwise, the income, education, and position should be included separately.**
  We agree, education is no longer included.
- **Nothing was reported about vitamin supplement usage.**
  We added the following to the limitations:
  Hcy levels of participants might be influenced by the supplementary intake of vitamins. We were not able to account for supplementary vitamins in our analysis.
- **2- The study aims at studying the association between Hcy (exposure) and myopia (outcome). It is understandable that participants with missing data on exposure or outcome are excluded. However, it is not clear why to exclude 1322 subjects with a history of cancer and 951 with previous ocular surgery? Conditioning on these two diseases is not justifiable. At least this should be justified and considered in the sensitivity analysis.**
  We revised the inclusion and exclusion criteria, as well as the selection of potential confounders:
  “Exclusion criteria were insufficient knowledge of German and physical or mental inability to participate in the examinations in the study center. […] For the analysis of refraction, we included all participants with available information on Hcy and refraction at baseline and with phakic lens status and no history of corneal surgery or corneal laser treatment. For the analysis of 5-year change in refraction, we further excluded all participants without Hcy or refraction measurements at the 5-year follow-up examination and with lens replacement surgery, corneal surgery or corneal laser treatment between baseline and follow-up examination.”
  “Model 1 included no covariates, model 2 was adjusted age, sex and socio-economic status. For the identification of potential confounders we followed the directed acyclic graph (DAG) concept [16]. We used the online tool DAGitty V2.3 (http://dagitty.net/, last accessed 2019-08-16) to visualize potential causal relationships (Supplementary Figure S) and to derive the minimal sufficient adjustment set for estimating the total effect of Hcy on refraction [17].”
- **3- Table 1, is not showing interquartile range as stated in the footnote. It could be the 25-75th percentiles; the IQR is one value, or the distance between the two percentiles.**
  We corrected our description:
  “For continuous variables, we calculated median and 25th and 75th percentiles, and mean and standard deviation for approximately normal distributed variables.”
- **4- Why not using the same way of data expression (Median, IQR) for all variables instead of mixing means and medians?**
  As far as I know, this is the way it is recommended, and it was used in all previous GHS publications.
- **5- Table 1 should include an additional column to report the p values for the differences between men and women.**
  Since our primary research question is not to explore sex differences, I would prefer not to report p values for the differences. Besides that, I do not see the increase in information by reporting p values in addition to the summary values. If there is a clinical relevant difference, it could be spotted anyway. On the other hand, even small differences are likely to get flagged with a low p value, given our large sample size.
- **6- Table 2, same comment on interquartile range. This is not interquartile range.**
  OK, please see above.
- **7- Table 2 should report the p values for the difference between men and women.**
  Please see above.
- **8- Table 2: It is not clear how the scoring of myopia was performed, since the next table includes the number of eyes. Presumably the study did not differentiate between subjects having myopia in one eye or in two eyes in the same time. Accurate measurement of the outcome is essential, since having two affected eyes is more severity than having only one eye.**
  We updated our categorization of myopia (and tables), but used the refraction (of both eyes) as a continuous in our regression models.
- **9- It is also not clear why the authors reporting mean refraction (mean SE of both eyes)? Line 144.**
  We used mean spherical equivalent for descriptive tables and figures, but our analyses are based on the refraction of both eyes:
  “We used linear regression models with general estimating equations for consideration of the correlation between fellow eyes to assess the association of Hcy and refraction and change in refraction [16].”
- **10- Principally, all tables should cite in the footnote the tests applied to analyze the dat. Looking at table 3 for instance, it is not easy to understand what are the reported numbers without going back to the test and data analyses.**
  We improved tables and footnotes.
- **11- Table 3 and 4 give the impression that the data analysis was not thoughtfully planned. The tables are citing now physical activity as a confounder. What is the rational for this and why this was not introduced in the part dealing with co-variates. Why should physical activity confound the association between hcy and myopia?**
  We updated the confounder selection:
  “Model 1 included no covariates, model 2 was adjusted age, sex and socio-economic status. For the identification of potential confounders we followed the directed acyclic graph (DAG) concept [15]. We used the online tool DAGitty V2.3 (http://dagitty.net/, last accessed 2019-08-16) to visualize potential causal relationships (Supplementary Figure S) and to derive the minimal sufficient adjustment set for estimating the total effect of Hcy on refraction [16].”
- **12- Where relevant (example figures), homocystein should be homocysteine.**
  ok
- **13- The conclusion of the study is deficient and not reflecting the data precisely. Sensitivity analysis and a threshold analysis shou [truncated, additional comments received from reviewer 2 below, upon request via PLOS ONE]**
- **Sensitivity analysis and a threshold analysis should be included. Sensitivity analysis includes many possibilities that could have caused biased results or conclusions. Sensitivity analysis should be indeed planed in the data analysis phase. Examples could be: data anaylsis according to sex; subgroup analysis considering the threshold (eg. thcy > 14 micromol/L); re-considering exclusion criteria; subgroups according to age, etc. '**
  We decided to use both outcome and exposure as acontinuous variable in our analysis, since dichotomizing would have resulted in a loss of power.
  To explore a potential threshold effect (i.e. association of homocysteine with refraction only if homocysteine is elevated above a threshold), we repeated the analyses limited to participants with Hcy >15 mmol/l.
  We are aware of the sex differences in Hcy levels, and sex was considered as a potential confounder. In addition, we repeated the analyses split by sex.
  These sensitivity analyses lead to similar results and are presented in the supplement.
- **The conclusion of the study is deficient and not reflecting the data precisely. The authors wrote:
  “We found no evidence for an association of minor elevations in Hcy with myopia or 5-year changes in refraction in this large population based cohort.”
   However, on the one hand it is not clear what “minor elevation of tHcy is meant to be” since tHcy is analysed as a continuous variable and its concentrations were not elevated in the majority of subjects. Moreover, the conclusion is too definitive for the cohort as if this is the final evidence. The conclusion should in my opinion leave a roam for the possibility that principally the hypothesis was tested in the wrong population (without HHCY, with a broad age range, and with many exclusion criteria that are not justifiable).**
  We rephrased the conclusion, but partly disagree with the reviewer: We had a considerable amount of participants with elevated Hcy, therefore some variance in the exposure. This provided us the opportunity to explore our research question. We acknowledge the criticism of our exclusion criteria and revised them accordingly, as described above.
- **The same problem again in the last paragraph of the discussion. The authors wrote:
  “We found no association between Hcy and myopia and so no evidence for a clinically significant contribution of minor elevations in Hcy to the risk of myopia.”
  Also here, the conclusion is too definitive, despite that the major limitation is that the population has indeed no hyperhomocysteinemia. Minor elevation in Hcy is not defined, and indeed this global expression (minor elevations in Hcy) does not mean anything without defining a quantitative threshold and data showing this.**
  I do not completely agree. The majority of our sample had no hyperhomocysteinemia , as expected in a population-based sample. Therefore, our sample perfectly fits to our question of interest, namely to explore if minor elevations (below clinically significant hyperhomocysteinemia ) might contribute to the risk of myopia. In addition, we had some variation of Hcy in our sample, as now added to the results section:
  “86 % (n=11,814) had normal Hcy levels (<= 15 μmol/l), 13 % (n=1840) mild hyperhomocysteinemia (>15 to <=30 μmol/l), 0.7 % (n=91) intermediate hyperhomocysteinemia (> 30 to <= 100 μmol/l), and n=4 serious hyperhomocysteinemia (> 100 μmol/l).”
